# Chiari Osteotomy in the Children: Mid-term Follow-Up Results

**DOI:** 10.7759/cureus.56053

**Published:** 2024-03-12

**Authors:** Nabil Chettahi, Mohammed Tazi Charki, Othmane Rais, Hicham Abdellaoui, Karima Atarraf, Moulay Abderrahmane Afifi

**Affiliations:** 1 Department of Pediatric Surgery, Centre Hospitalier Universitaire Hassan II, Sidi Mohamed Ben Abdellah University, Féz, MAR; 2 Department of Traumatology and Pediatric Orthopedics, Centre Hospitalier Universitaire Hassan II, Sidi Mohamed Ben Abdellah University, Féz, MAR

**Keywords:** bone and joint diseases, hip dysplasia, chiari osteotomy, hip joint, pelvic osteotomy

## Abstract

Introduction

The Chiari osteotomy enlarges the acetabulum to increase coverage of the femoral head. It is performed as a salvage procedure on a noncongruent, yet in-place, hip. This study aims to assess the clinical and radiographic outcomes of Chiari pelvic osteotomy for treating hip dysplasia in children.

Methods

This is a case series conducted in the pediatric orthopedic trauma department of the Centre Hospitalier Universitaire Hassan II, Fez, Morocco, over a 10-year period from January 2011 to December 2020. The study included patients who were being treated for hip dysplasia and had undergone a Chiari osteotomy. Two types of assessments were used to evaluate global hip function: a clinical assessment using the Merle d'Aubigné and Postel score, and a radiological assessment involving measurements taken from frontal pelvic radiographs.

Results

A total of 12 Chiari osteotomies were performed in nine patients. The mean age at surgery was 10.8 ± 1.7 years and the mean follow-up was 4.6 ± 2.78 years. The clinical assessment score improved statistically during the last follow-up compared with the preoperative measurements for pain (p< 0.001), mobility (p = 0.002), walking (p<0.001), and total score (p< 0.001), for which 3.8 ± 1.9 points could be gained. Surgically, the osteotomy line height was 5.4 ± 2.6 mm, the osteotomy angle was 12.5 ± 2.2°, and the translated distance was 18.5 ± 3.2 mm. Regarding radiological evaluation, the comparison of angle measurements between preoperative and final recoil was statistically significant for both the vertical center edge (VCE) angle (p<0.001) with a mean gain of 16.33 ± 4.79° and the high transverse edge (HTE) angle (p = 0.002) with a mean loss of 12.67 ± 10.88°.

Conclusion

Chiari pelvic osteotomy is a complex procedure that requires very precise techniques. However, it results in remarkable relief for patients, providing an immediate impact on the Merle d'Aubigné and Postel score, particularly with regard to pain.

## Introduction

Hip dysplasia is characterized by a deformity in the hip joint that causes inadequate coverage of the femoral head, leading to joint instability [1]. Over time, this instability can lead to various anatomical changes in the joint, which may cause pain and speed up the onset of hip osteoarthritis [2].

Managing hip dysplasia in older children poses unique challenges compared to treatment in younger patients due to the specific anatomical complexities of the hip joint at an older age [3]. To address these challenges and provide more effective treatment, surgical interventions such as Chiari pelvic osteotomy have been developed.

Karl Chiari first introduced the Chiari pelvic osteotomy in 1953 as a surgical procedure intended to salvage compromised coxofemoral joints. The primary goals of this surgery are to extend the joint's lifespan and improve its function by reducing the mechanical load exerted on each unit area of the joint [4].

During this surgical procedure, a pelvic osteotomy is performed on the ilium, specifically targeting the anterior inferior iliac spine located just above the joint capsule's attachment point. This involves two key displacements: moving the distal segment of the ilium medially and shifting the proximal segment laterally. These manipulations create a new "roof" over the femoral head, thereby improving its coverage. The joint capsule is interposed between the acetabulum and the femoral head, generally leading to the satisfactory formation of a new acetabular surface [5].

The aim of this study was to assess the clinical and radiographic outcomes of Chiari pelvic osteotomy in treating hip dysplasia during a mid-term follow-up. 

## Materials and methods

This retrospective case series was carried out in the Pediatric Orthopedic Traumatology Department of Centre Hospitalier Universitaire Hassan II in Fez, Morocco, over a 10-year period, from January 2011 to December 2020. It included pediatric patients diagnosed with hip dysplasia who had undergone Chiari osteotomy surgery due to severe dysplasia and pain. Patients who were lost to follow-up or had a follow-up period of less than six months were excluded from the study in order to ensure robust data analysis. Data was gathered using a standardized operating sheet, which recorded the personal, clinical, and radiological details of each patient.

Evaluation metrics

Two methods were employed to assess the overall function of the hip joint.

Clinical Assessment

The patients underwent clinical assessment using the Merle d'Aubigné and Postel Method, which evaluates pain, gait, and mobility on a scale of 1 to 6 for each item. A score of 1 indicates the worst state while a score of 6 represents the best state for the patient. The total minimum achievable score is 3, with a maximum possible score of 18.

Radiological Evaluation

Radiological evaluation was done utilizing a frontal pelvis radiograph to measure the following angles: (i) Vertical center edge (VCE) angle (Wiberg’s angle), which measured the coverage of the femoral head by the acetabulum; (ii) High transverse edge (HTE) angle, which measured the orientation of the acetabulum in relation to the pelvic plane. Evaluations occurred at three different times: before the surgery, after the surgery, and during the final follow-up.

Surgical approach and procedure details

The Smith-Petersen approach, also known as the direct anterior approach, was used. The height of the osteotomy line was set at 5.2 mm with a range of 3−12 mm, and its angle was 12.5∘ with a range of 8-15∘. The translated distance was 18.5 mm within a range of 15−25 mm. To control the acetabulum's medialization, traction and abduction were applied to the lower limb. For fixation, we conducted five osteotomies using screws, along with four using K-wires, and another three without any fixation.

Statistical analysis

Descriptive statistics were used to summarize the demographic, clinical, and radiological characteristics of the participants. Frequencies were utilized for qualitative variables, while means and standard deviations were employed for quantitative variables. We used a matched-sample student's t-test to analyze the clinical and radiological changes in our patients, with a significance level set at 5%. All statistical analyses were performed using R software (R Foundation for Statistical Computing, Vienna, Austria).

## Results

Patient demographics

A total of 12 Chiari osteotomies were performed across nine patients, with three bilateral procedures and six unilateral ones. The average age of patients at the time of the operation was 10.8±1.7 years. The mean follow-up duration was 4.6±2.78 years (Table 1).

**Table 1:** Patient characteristics Data given in mean (SD) and n (%), as indicated.

Characteristics		Values
Age of operation (years), mean (SD)		10.83 (1.7)
Current age (years), mean (SD)		15.42 (4.1)
Follow-up (years), mean (SD)		4.6 (2.78)
Sex, n (%)	Female	8 (66,7)
	Male	4 (33,3)
Operated side, n (%)	Right	7 (58.3)
	Left	5 (41.7)

Clinical evaluation

**Figure 1:**
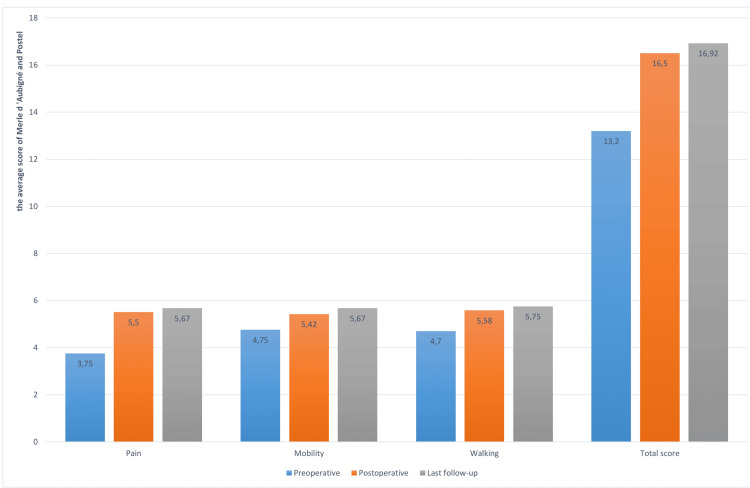
Clinical evaluation according to the Merle d'Aubigné-Postel score

For clinical assessments (Figure 1), the averages of the Merle d'Aubigné-Postel score were: Preoperatively, the pain score was 3.75±1.14, mobility was rated at 4.75±0.95, and walking scored an average of 4.67±0.49; Postoperatively, the scores were 5.5±0.7 for pain, 5.42±0.7 for mobility, and 5.6±0.5 for walking; and at the last follow-up, the scores were 5.67±0.49 for pain, 5.67±0.49 for mobility, and 5.75±0.49 for walking.

The mean score improvements were 1.9 ± 1.2 for pain, 0.9 ± 0.8 for mobility, and 1.1 ± 0.3 for walking, with a significant total score improvement of 3.8 ± 1.9 (Table 2).

**Table 2:** Analysis of the clinical evolution of our patients

	Preoperative	Last follow-up	p-value
Pain, mean (SD)	3.75 (1.14)	5.67 (0.49)	< 0.001
Mobility, mean (SD)	4.75 (0.95)	5.67 (0.49)	0.002
Walking, mean (SD)	4.67 (0.49)	5.75 (0.45)	< 0.001
Total score, mean (SD)	13.17 (2.13)	16.92 (0.99)	< 0.001

The limb length discrepancy was 1.17±0.65 cm before surgery and increased to 1.63±0.77 cm at the last follow-up, with an average growth of 0.46±0.39 cm.

Radiological evaluation

**Figure 2:**
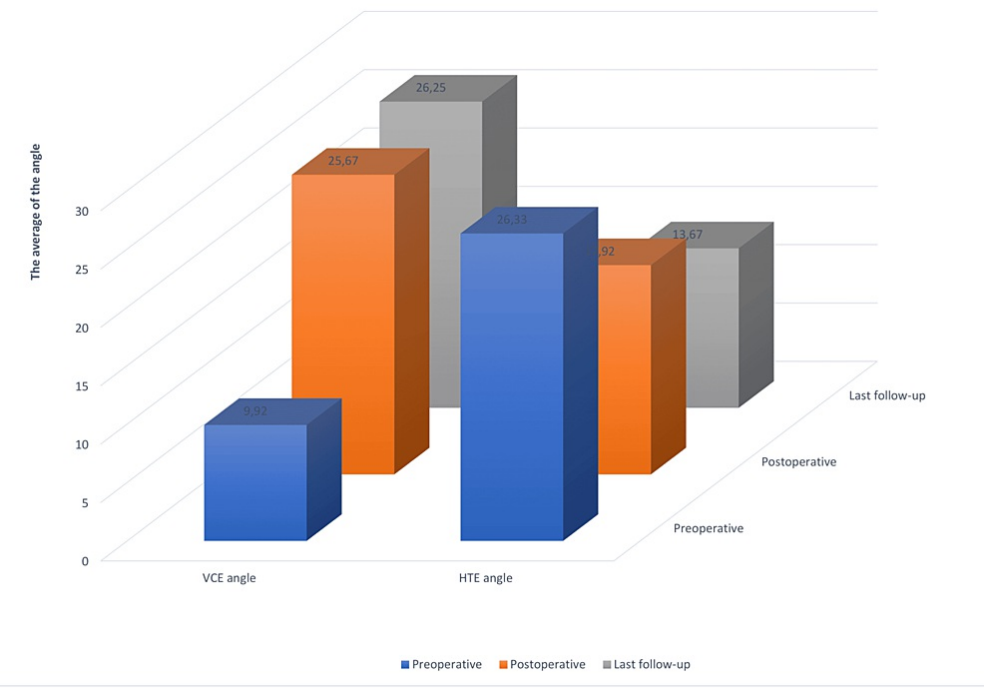
Radiological evaluation

Radiological assessments (Figure 2) revealed the following angle averages: Preoperatively, 9.92±4.99^∘^ for the VCE angle and 26.33±12.53^∘^ for the HTE angle; Postoperatively, 25.67±4.94^∘^ for the VCE angle and 17.92±7.19∘ for the HTE angle; and at the last follow-up, 26.25±5.26^∘^ for the VCE angle and 13.67±4.44^∘^ for the HTE angle.

The average gains were 16.33±4.79^∘^ for the VCE angle and a loss of 12.67±10.88^∘^ for the HTE angle. These changes were statistically significant (Table 3).

**Table 3:** Analysis of the radiological evolution of our patients VCE: vertical center edge; HTE: high transverse edge

	Preoperative	Last follow-up	p-value
The VCE angle (Wiberg’s angle), mean (SD)	9.92 (4.99)	26.25 (5.26)	< 0.001
The HTE angle, mean (SD)	13.67 (4.44)	26.33 (12.53)	0.002

## Discussion

The primary objective of our study was to assess the outcomes of the Chiari osteotomy in treating hip dysplasia in children and interpreting the mid-term follow-up results. We were able to ascertain that this surgical approach yields notably favorable results, both clinically and radiologically.

The Chiari osteotomy we used is a salvage procedure. It leverages the cancellous part of the ilium and the interposed hip joint capsule to encase the femoral head, aiding in weight-bearing. Salvage procedures aim to reduce pain, delay impending arthroplasty, and enhance function during this time. Fabula et al. noted that salvage techniques like the Chiari osteotomy are most effective for younger patients with incongruent hips and limited remodeling potential [6]. On the other hand, reconstructive procedures are more suitable when the cartilage remains healthy and the joint is congruent. These factors should be considered when deciding on a surgical approach for pediatric patients with hip dysplasia.

Our study showed significant improvements in various aspects of the Merle d'Aubigné and Postel score, with an average follow-up period of over four years following the Chiari osteotomy. Notably, the most substantial improvement was seen in the pain metric, which had initially displayed the lowest preoperative score. This aligns with previous research suggesting that Chiari osteotomy often leads to better pain relief outcomes [7,8]. Furthermore, Bennett et al. documented complete pain relief in all pediatric patients who underwent Chiari osteotomy and were later clinically evaluated after an average of four years post surgery [9].

Regarding limb length discrepancy, our analysis revealed a minor shortening in the operated limb after the Chiari osteotomy. Our results are consistent with another study that found post-operative limb shortening did not exceed 1 cm [10,11].

Radiographically, we observed a significant improvement in angle measurements, with an increase in the VCE angle and a decrease in the HTE angle. These results are consistent with a study on Chiari osteotomy conducted on 22 children, averaging 8.5 years of age, which also showed considerable radiological improvement [12]. However, these findings differ from other studies that have criticized this surgical technique in children due to the anti-Chiari effect, having recorded unsatisfactory results in either all [13] or half [14] of their participants. Hosny et al. linked this anti-Chiari effect to age, noting unsatisfactory outcomes in 68% of patients under 10 years of age and only 23% among older age groups [15]. This disparity may be attributed to the significant growth potential of the acetabulum leading to femoral head decentering and superior shift in younger children [16].

In our study, we were unable to assess the progression to osteoarthritis because the average follow-up duration for patients was only 4.5 years, which is not sufficient to determine long-term evolution. However, studies have suggested that Chiari osteotomy can remain radiologically effective for an extended period of 25-30 years [17,18].

Chiari osteotomy could significantly affect the subsequent treatment plan for patients who may undergo total hip arthroplasty. According to Hashemi-Nejad et al., there were superior clinical and radiographic outcomes after total hip arthroplasty (THA) in patients who had previously undergone a successful anterior Chiari osteotomy compared to those in a control group without prior pelvic osteotomy [19]. It was observed that THA in the post-Chiari cohort required fewer acetabular augmentation procedures, had shorter operative times, minimal blood loss, and fewer early-stage complications.

Our study was significantly limited by the absence of Lequesne's false profile radiograph of the hip, which prevented us from calculating the anterior acetabular cover angle both preoperatively and during follow-up. However, our study still holds importance due to its strong clinical and radiological outcomes.

## Conclusions

Our study shows that the Chiari osteotomy is an effective surgical approach for treating hip dysplasia in pediatric patients. Our mid-term follow-up findings consistently demonstrated positive clinical and radiological outcomes after the procedure. There are improvements in pain relief, mobility, and walking, along with significant radiological changes. However, further long-term studies are necessary to fully understand its lasting efficacy and potential implications in managing hip dysplasia.
